# Effects of Kasugamycin on the Translatome of *Escherichia coli*

**DOI:** 10.1371/journal.pone.0168143

**Published:** 2017-01-12

**Authors:** Christian Lange, Matthias Lehr, Karolin Zerulla, Petra Ludwig, Jens Schweitzer, Tino Polen, Volker F. Wendisch, Jörg Soppa

**Affiliations:** 1 Goethe University, Biocentre, Institute for Molecular Biosciences, Frankfurt, Germany; 2 University of Bielefeld, Faculty of Biology, University of Bielefeld, Bielefeld, Germany; Niels Bohr Institute, DENMARK

## Abstract

It is long known that Kasugamycin inhibits translation of canonical transcripts containing a 5’-UTR with a Shine Dalgarno (SD) motif, but not that of leaderless transcripts. To gain a global overview of the influence of Kasugamycin on translation efficiencies, the changes of the translatome of *Escherichia coli* induced by a 10 minutes Kasugamycin treatment were quantified. The effect of Kasugamycin differed widely, 102 transcripts were at least twofold more sensitive to Kasugamycin than average, and 137 transcripts were at least twofold more resistant, and there was a more than 100-fold difference between the most resistant and the most sensitive transcript. The 5’-ends of 19 transcripts were determined from treated and untreated cultures, but Kasugamycin resistance did neither correlate with the presence or absence of a SD motif, nor with differences in 5’-UTR lengths or GC content. RNA Structure Logos were generated for the 102 Kasugamycin-sensitive and for the 137 resistant transcripts. For both groups a short Shine Dalgarno (SD) motif was retrieved, but no specific motifs associated with resistance or sensitivity could be found. Notably, this was also true for the region -3 to -1 upstream of the start codon and the presence of an extended SD motif, which had been proposed to result in Kasugamycin resistance. Comparison of the translatome results with the database RegulonDB showed that the transcript with the highest resistance was leaderless, but no further leaderless transcripts were among the resistant transcripts. Unexpectedly, it was found that translational coupling might be a novel feature that is associated with Kasugamycin resistance. Taken together, Kasugamycin has a profound effect on translational efficiencies of *E*. *coli* transcripts, but the mechanism of action is different than previously described.

## Introduction

About 50 years ago it was discovered that a new isolate later named *Streptomyces kasugiensis* produced an antibiotic that was active against many different bacteria [1]. The new antibiotic was named Kasugamycin, because the producing strain had been isolated near the Kasuga shrine at Nara City in Japan. Kasugamycin has been applied in medicine to treat *Pseudomonas aeruginosa* infections and is still used in agriculture to fight plant pathogens [2–6].

Characterization of the Kasugamycin action revealed that it binds to the 30S ribosomal subunit and the 70S ribosome of *E*. *coli* with a 1:1 stoichiometry, but not to the 50S subunit. It was found to inhibit the initiation step of translation [7]. As it inhibits binding of the initiator tRNA (tRNA_i_) to the P-site (peptidyl site) of the ribosome, it was long thought that Kasugamycin also binds to the P-site and acts via competitive inhibition [7]. However, the structures of complexes of Kasugamycin with the *Thermus thermophilus* 30S subunit and with the *E*. *coli* 70S ribosome have been determined [8,9] and revealed that in both cases the Kasugamycin binding site did not overlap with the tRNA_i_ binding site. Instead, Kasugamycin bound further upstream, at the E-site of the ribosome, which is devoid of a tRNA during initiation. Kasugamycin blocked the normal path of the 5’-UTR of the mRNA upstream of the start codon, and in both cases it was concluded that the inhibitory effect on tRNA_i_ binding must be indirect. Notably, the localization of the Kasugamycin binding site nicely explained earlier observations that Kasugamycin inhibits translation initiation on canonical transcripts with a Shine Dalgarno motif in its 5’-UTR, but not on leaderless transcripts lacking a 5’-UTR [10]. It has been argued that two effects contribute to the lack of inhibition at leaderless transcripts: 1) obviously, in the absence of a 5’-UTR there is no steric hindrance between Kasugamycin and the mRNA, and 2) leaderless mRNAs need the 70S ribosome for translation initiation [11], and the presence of the 50S subunit strengthens tRNA_i_ binding in the P-site [9].

Several additional lines of evidence indicate that the mechanisms of translation initiation are drastically different for canonical transcripts with 5’-UTR and SD motif versus leaderless transcripts, e.g. 1) leaderless transcripts tolerate only very few nucleotides upstream of the start codon to allow efficient translation, both in *E*. *coli* [12] and the archaeon *Haloferax volcanii* [13,14], and thus the region of the SD motif is inhibitory for leaderless initiation, 2) leaderless transcripts need the undissociated 70S/80S ribosome [11,15], 3) the initiation factor dependence is different for canonical transcripts and leaderless transcripts [10,16,17], 4) the temperature-dependence of translation on both types of transcripts is different [16], and 5) and the start codon preference is different, again both in *E*. *coli* [18,19] and in *H*. *volcanii* [14].

Some species are known to make extensive use of leaderless transcripts, e.g. the lower eukaryote *Giardia lamblia* contains exclusively leaderless transcripts [20], 72% of the transcripts of haloarchaea is leaderless [13], and 33% and 47%, respectively, of the transcripts of the bacterium *Corynebacterium glutamicum* and *Deinococcus* deserti are leaderless [21–23]. The fraction of leaderless transcripts in *E*. *coli* was not known at the start of this project. It was known that only about 60–70% of all *E*. *coli* genes are preceded by a SD motif and follow the canonical model [24,25]. However, the transcripts of the other genes might be leaderless or might belong to another class of non-canonical transcripts, which contain a 5’-UTR that is devoid of a SD motif. As Kasugamycin does not inhibit translation on leaderless transcripts, the characterization of the translatome after treatment with Kasugamycin seemed to be an ideal approach for the enrichment and identification of cellular leaderless transcripts. Nearly all studies on the binding site of Kasugamycin or the molecular mechanism of its action have been performed with *E*. *coli*, therefore, also the current study also used this species. Other archaeal and bacterial species have higher fractions of leaderless transcripts, however, results obtained with these species could not have been compared to previous publications.

In addition, it has been revealed that the degree of inhibition of translation on leadered mRNAs is not identical, but that considerable differences exist [9]. It has been proposed that the—ill-defined—specific interactions of the respective mRNAs with the Kasugamycin-30S complex are responsible for the extent of inhibition, or even for the absence of inhibition for some examples of leadered transcripts. The region of -3 to +1 was predicted to be of specific importance for the inhibitory effect of Kasugamycin on leadered transcripts. In addition, it has been proposed that the inhibitory effect of Kasugamycin on leadered transcripts is smaller when the 5’-UTR contains a strong SD motif [8]. Therefore, a genome-wide quantification of the inhibitory effect on the translatome of *E*. *coli* aimed at identification of mRNA features that make transcripts especially sensitive and especially resistant, respectively, to the inhibition by Kasugamycin. Especially, the approach should clarify whether a correlation exists between the presence of a strong SD motif and a high resistance to Kasugamycin. To this end, the 5’-ends of 19 transcripts from treated and from untreated cells have been determined, bioinformatics analyses of the 5’-UTRs from the groups of sensitive and resistant transcripts have been performed, and the translatome results have been compared with RegulonDB.

## Results

### The effect of Kasugamycin on the growth of *E*. *coli*

**Fig 1:**
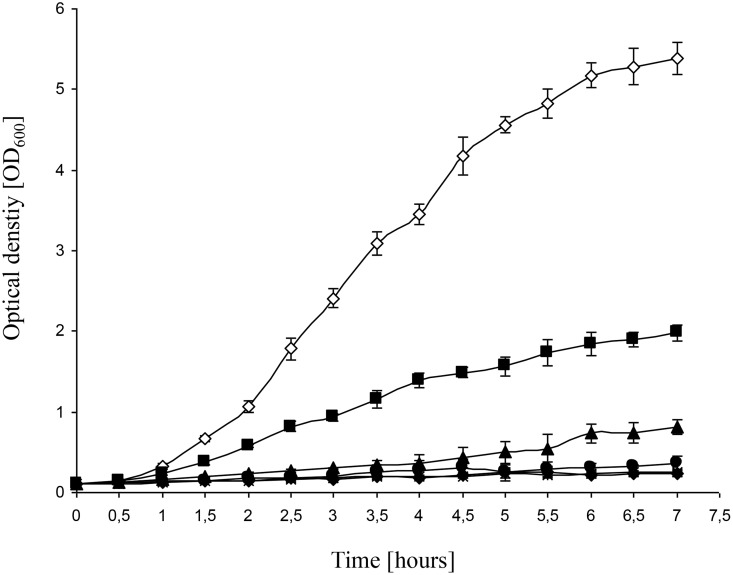
Inhibitory effect of Kasugamycin on the growth of *E*. *coli* MG1655. Cultures were grown in the presence of different Kasugamycin concentrations, and the optical densities were determined at the indicated times. Three biological replicates were grown, and average values and standard deviations are shown. Empty diamonds: control without Kasugamycin; filled squares: 100 μg/ml; filled triangles: 250 μg/ml; filled circles: 500 μg/ml; asterisks: 750 μg/ml; filled diamonds: 1000 μg/ml.

The *E*. *coli* strain MG1655 was used for the present study, because it is widely used, its genome has been sequenced [26], and it is often regarded as a “wild-type” strain in spite of several mutations. First, the concentration dependence of Kasugamycin inhibition was determined. Fig 1 shows the growth of MG1655 in the presence of various Kasugamycin concentrations, and it was revealed that the minimal inhibitory concentration is 500 μg/ml. This is a higher sensitivity than has been reported for the *E*. *coli* strain W3110, for which a concentration of 750 μg/ml did not inhibit growth totally, but only reduced the growth rate by a factor of five [27]. To be on the safe side, it was decided to use a concentration of 750 μg/ml for all following experiments.

### The effect of Kasugamycin on the translatome of *E*. *coli*

**Fig 2:**
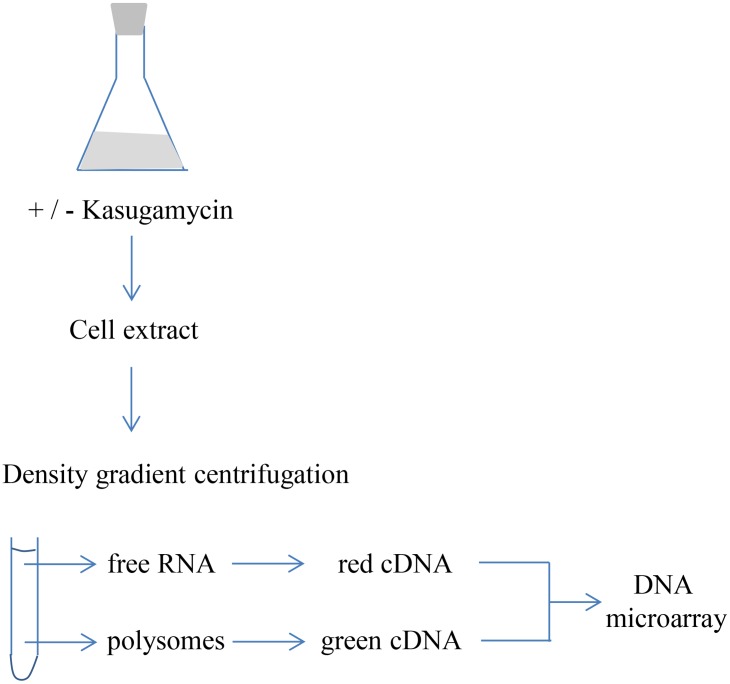
Schematic overview of a translatome experiment.

Each translatome analysis was started with one fresh MG1655 colony. Cultures were grown exponentially from OD_600_ of 0.02 to OD_600_ of 0.2. At this point, 30 ml were removed as a non-treated control, and Kasugamycin was added to the remaining 130 ml. After 10 minutes cultivation in the presence of Kasugamycin, 30 ml were removed to yield the Kasugamycin-treated sample. Cells of the treated cultures and the untreated controls were harvested by centrifugation. After resuspension of the cell pellets, cells were lysed by sonication, and cell debris was removed by centrifugation. Density gradients were used to separate a fraction containing free mRNAs, which were not translated at the time of cell harvesting, and a fraction containing polysomal mRNAs, which were actively translated at the time of cell harvesting. Both fractions were used to isolate RNA, to generate fluorescently-labeled cDNA, and to compare the translation status of all mRNAs by competitive hybridization using an *E*. *coli* DNA microarray. Fig 2 gives an overview of the experimental approach of the translatome analysis. Five independent biological replicates were analyzed for the untreated control as well as for Kasugamycin-treated samples.

The DNA microarray was comprised of probes for 4382 genes. The analysis was restricted to transcripts that yielded results in at least three of the five biological replicates in the untreated as well as the treated cultures, which was the case for 2801 genes. All results are summarized in S1 Table. The relative average translational efficiencies and their standard deviations were calculated for the 2801 transcripts in the untreated and treated cells (ratio of ribosome-bound versus free mRNA). In addition, the variation coefficients were calcutated (quotients of standard deviations and average translational efficiencies). High variations can have different reasons, therefore, all results with high variation coefficients (60% or more) were inspected individually. When high variations were caused by obvious outliers, these values were excluded from the analysis (red values in S1 Table). Lastly, the degree of inhibition by Kasugamycin was calculated as the ratio of the relative translational efficiencies in the treated culture divided by the relative translational efficiencies of the untreated culture, and the resulting values were named “Kasugamycin effect values” (KEVs). Because the translatome results of the untreated and the treated culture had been normalized, a value of one does not mean that Kasugamycin does not have any effect on translation, but it represents the average Kasugamycin effect. A KEV below 1 represents an over-average degree of inhibition by Kasugamycin and the transcripts were termed “sensitive”, a KEV above 1 represents an under-average inhibition (and not a stimulation by Kasugamycin) and the transcripts were termed “resistant”. Of course the differences between treated and untreated samples (KEV values) have different significanes for the 2801 genes, e.g. because the number of analyzable replicates varied. To address this quantitatively, a statistical t-test was performed for all 2801 genes, and the resulting p-values are also included in S1 Table.

**Fig 3:**
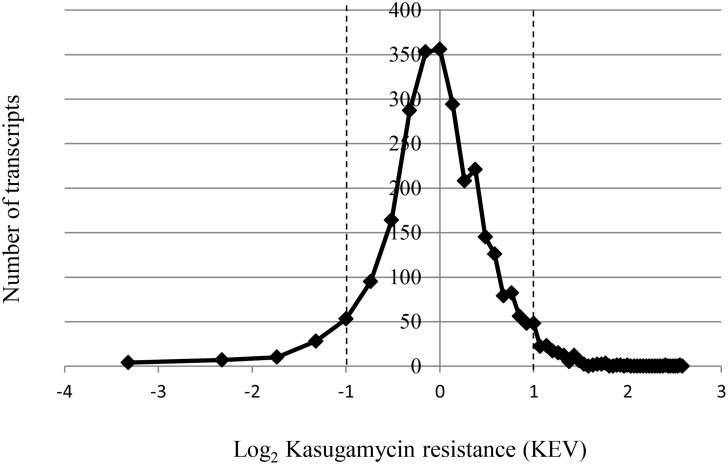
The distribution of Kasugamycin resistance in the translatome of *E*. *coli*. The KEV value representing a quantitative value of the degree of Kasugamycin resistance was calculated for each of the 2801 analyzed *E*. *coli* transcripts as explained in the text. The KEV values were transformed into log_2_ values, and the number of transcripts was plotted against the resistance.

The KEVs were transformed into log_2_ values, and the distribution is shown in Fig 3. A normal distribution around the average value was obtained. An at least twofold deviation from the average was defined as threshold, and thus transcripts with KEVs of 0.5 or lower were regarded as sensitive (log_2_ < -1) and transcripts with KEVs of 2.0 or higher were regarded as resistant (log_2_ >1) to Kasugamycin, respectively. Using this definition, 102 of the analyzed 2801 transcripts were Kasugamyin sensitive, while 137 of the 2801 transcripts were Kasugamycin resistant. The degree of inhibition of Kasugamycin varied considerably: the translational efficiency of the most resistant transcript was 100-fold higher than that of the most sensitive transcript. As described in the folloging paragraphs, we used several approaches to unravel systematic differences between the resistant versus the sensitive transcripts, which could be informative about the Kasugamycin function.

### Analysis of 5’-UTRs of selected sensitive and resistant transcripts

Ten sensitive transcripts with KEVs from 0.48 to 0.25 and ten resistant transcripts with KEVs from 2.14 to 5.21 were chosen and their 5’-ends were determined by 5’-RACE using untreated cultures as well as cultures after a 10 minutes Kasugamycin treatment. In one case the 5’-end determination was unsuccessful, the results of the remaining 22 transcripts of 19 genes are summarized in Table 1 together with relevant features, e.g the presence of a SD motif and its distance from the start codon. In all cases the 5’-ends were identical in treated and untreated cultures. The small difference of 31 nt to 28 nt of the *dnaB* transcript was regarded as insignificant and both represent the same transcript. It has been reported that a Kasugamycin treatment of 2 hours results in a change of transcript 5’-ends in some cases [28]. We could not find any indication for an influence of Kasugamycin on the 5’-end after a ten minutes treatment, neither for sensitive nor for resistant transcripts.

**Table 1:** Transcripts with experimentally-determined 5'-ends and selected features.

b-No.	Gene	KSG effect [KEV]	5'-UTR - KSG [nt]	5'-UTR + KSG [nt]	Distance to AUG [bp]	SD-motif	Sequence at SD site^1^
b1809	*yoaB*	0,25	31	31	5	yes	**T**G**AGGA**CA
b1238	*tdk*	0,34	34	34	3	no	GCCT**G**T**GG**
b3180	*yhbY*	0,36	31	31	7	yes	**TAAG**C**A**AA
b1779	*gapA*	0,37	36	36	5	no	GCT**GG**T**GG**
b2765	*sscR*	0,37	24	24	4	no	**T**GTA**GAG**A
b1824	*yobF*	0,39	27	27	5	no	**TA**CA**G**TTC
b2412	*zipA*	0,40	47	47	6	no	C**AA**CAGA**G**
b3357	*crp*	0,45	166	166	7	yes	AG**AGGA**TA
b3357	*crp*		68	68			
b0762	*ybhT*	0,47	193	193	5	yes	**T**CT**GGAG**T
b0762	*ybhT*		22	22			
b0947	*ycbX*	0,48	25	25	5	yes	**T**G**AGGA**CC
b2682	*ygaZ*	2,38	48	48	6	yes	**TAAG**CGTA
b0882	*clpA*	2,56	53	53	5	yes	GGG**GGAGG**
b0882	*clpA*		173	173			
b4052	*dnaB*	2,61	31	28	6	no	**TAA**CTCCA
b0081	*mraZ*	2,69	45	45	5	yes	GGGT**GAGG**
b3958	*argC*	2,74	118	118	4	no	**T**G**A**AT**AG**C
b0850	*ybjC*	3,07	21	21	7	yes	**T**GCA**GAGG**
b2208	*napF*	3,61	77	77	6	yes	G**A**T**GGA**A**G**
b3321	*rpsJ*	3,79	20	20	5	no	G**A**GCTCT**G**
b3179	*rrmJ*	5,21	67	67	6	no	C**A**T**GG**GAA
**SD consensus**							**TAAGGAGG**

^1^the nucleotides that match the SD consensus are shown in bold

Comparison of the 5’-UTRs of the sensitive and the resistant transcripts did not lead to the identification of any systematic difference. None of the 22 transcripts was leaderless, therefore, the Kasugamycin treatment had not led to the expected exclusive enrichment of leaderless transcripts in the group of resistant transcripts. There was no obvious difference in GC content, AU-rich motifs, etc. A SD motif was present in the 5’-UTRs of 5 out of 10 sensitive transcripts and in 5 out of 9 resistant transcripts. The average distance between the SD motif and the start codon was 5.8 nt for the sensitive as well as for the resistant transcripts. The 5’-UTR length varied from 22 nt to 193 nt for the sensitive transcripts and 20 nt to 173 nt for the resistant transcripts. For two sensitive transcripts and one resistant transcript two different 5’-ends were obtained. In all three cases one of the two alternative 5’-UTRs was very long with more than 150 nt. The most probable explanation is that these three genes are preceded by two promoters, alternatively, the shorter transcript might have been generated from the longer transcript by processing. It has been observed before that *E*. *coli* genes can be preceded by two (or more) promoters, and it has been estimated that this is true for about 20% of all genes [29]. Taken together, 5’-end determination and 5’-UTR comparison of 22 transcripts of 19 genes did not unravel any systematic differences between sensitive and resistant transcripts.

### Bioinformatic analyses of sensitive and resistant transcripts

**Fig 4:**
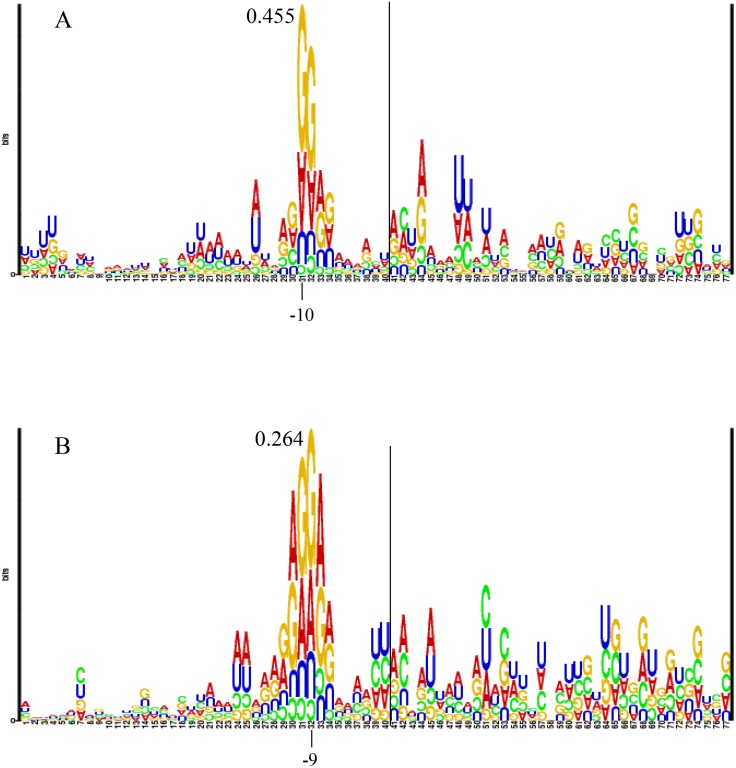
RNA Structure Logos of the groups of Kasugamycin sensitive and Kasugamycin-resistant genes. For the respective genes 40 nt upstream of the start codon and the first 40 nt of the open reading frames were retrieved from the database EcoGene3. The start codons were removed and the sequences were used to generate RNA Structure Logos at http://www.cbs.dtu.dk/~gorodkin/appl/slogo.html. RNA Structure LOGOS show the deviation of the occurrences of the four nucleotides at any position of a multiple sequence alignment from the statistical expectations based on the respective fractions of the four nucleotieds of the input sequences. The height of the nucleotides corresponds to the degree of deviation. Upright nucleotides have a higher occurrence as expected, inverted nucleotides a lower occurrence. A line indicates the border between the 40 nt upstream and the 37 nt downstream of the start codon. The information content (in bits) of the highest position is indicated **A.** LOGO of 102 Kasugamycin-sensitive genes (KEVs of < 0.5 in S1 Table). **B.** LOGO of 137 Kasugamycin-resistant genes (KEVs > 2.0 in S1 Table).

Another approach to identify systematic differences between sensitive and resistant transcripts was a bioinformatics analysis of all transcripts of the two groups. To this end, the regions around the start codons from -40 to + 40 were extracted for all 102 genes with KEV of lower than 0.5 and the 137 genes with KEV of higher than 2.0. RNA Structure LOGOs were generated for both groups of genes with the aim to detect signatures for Kasugamycin resistance and/or sensitivity. RNA Structure LOGOS compare the occurrences of the four nucleotides at each position of multiple sequence alignments with the statistical expectations based on the fractions of the four nucleotides in the input sequences. The height of the bases corresponds to the degree of deviation from the statistical expectations. The bases are shown upright when the occurrence is higher than the statistical expectation, and inverted when the occurrence is lower than the statistical expectation. The LOGO for the more than twofold sensitive genes is shown in Fig 4A, the LOGO for the more than twofold resistant genes is shown in Fig 4B. In both cases the SD motif was retrieved, with slight differences. The motif GGAG was retrieved for the sensitive genes at positions -10 to -7, while the motif GAGGAA was retrieved for the resistant genes from -12 to -7. Thus the SD motif is somewhat more extended for the resistant genes, in accordance with the prediction that a strong SD leads to higher resistance [8]. However, in both cases the information content is very small, and it is even smaller for the resistant genes (0.27 bit for G at -9) than for the sensitive genes (0.46 bit for G at -10). Thus, while the SD motif can be retrieved in both groups of genes, it is not a highly conserved determinant of any of the two groups and also not suitable to differentiate between sensitive and resistant genes. As controls, RNA Structure LOGOs were also calculated for three further group of genes, 1) 100 “rather sensitive” genes with KEV values of 0.15–0.61 (S1 Fig), 2) all 97 “very average” genes with KEV values of 0.99–1.01 (S2 Fig), and 3) 100 “rather resistant” genes with KEV values of 1.80–1.99 (S3 Fig). The three LOGOs look very similar, in each case a short SD motif was retrieved with a low information content of around 0.5 bits.Therefore, the RNA structure LOGOs indicated that a correlation between the presence of a SD motif and the degree of Kasugamycin resistance does not exist, Furthermore, they did not reveal any other motif that could differentiate between Kasugamycin resistant, average, and sensitive genes. Notably, this is also true for the positions -3 to -1, which had been predicted to be crucial for Kasugamycin resistance.

In an attempt to identify possible important motifs that do not have a fixed distance to the start codon, the program MEME was used to analyze both groups of genes. Unfortunately, no convincing motif that is present in a non-negligible fraction of genes in any of the two groups could be detected. The inspection of the functions of the sensitive and resistant genes in S1 Table also did not lead to the recognition of an over-average accumulation of specific biological functions in any of the two groups. Taken together, bioinformatics analyses of the groups of sensitive and resistant clones could not uncover any motif that might discriminate between them.

### Comparison of the results with the database RegulonDB

As an alternative approach to unravel transcript features possibly involved in Kasugamycin resistance or sensitivity, the results of the translatome study were compared to the database RegulonDB [30]. Features of possible relevance were retrieved for all sensitive and resistant transcripts that are listed in RegulonDB, and the results are summarized in S2 and S3 Tables.

Remarkably, the transcript with the highest Kasugamycin resistance, the *torS* transcript, has only 5 nt upstream of the start codon and is thus leaderless (S3 Table). However, it is the only example of the 137 resistant transcripts that is listed as being leaderless in RegulonDB. In contrast, four of the 90 sensitive transcripts, *yoaB*, *ycaM*, *yieK*, and *ynfA*, are listed to be leaderless in RegulonDB (S2 Table). However, RegulonDB lists two or more different 5’-ends for these four transcripts, making it impossible to correlate the observed Kasugamycin sensitivity to a specific leaderless or leadered version. Nevertheless, it is obvious that the 137 resistant genes do not contain a high fraction of leaderless transcripts.

Next, the sequences of the 5’-UTRs were analyzed, but no correlation between sequence, GC content, or presence of a SD motif with the degree of Kasugamycin resistance could be observed. The average length of 5’-UTRs of resistant genes was somewhat larger than that of sensitive genes (Table 2), but both groups of genes contained very short to very large 5’-UTRs (S2 and S3 Tables). Therefore, it might be that the fraction of structured 5’-UTRs is somewhat higher in the group of resistant genes and structured 5’-UTRs have a higher resistance, but the difference might as well be a random effect due to the given lengths of 5’-UTRs of transcripts that are sensitive or resistant for other reasons.

**Table 2:** Summary of selected features extracted from RegulonDB, which exhibit differences for the groups of Kasugamycin-sensitive versus—resistant genes. The number of genes that exhibit the respective feature are listed.

Feature	Sensitive	Resistant
No. genes in group	102	137
Not in RegulonDB	28	32
Listed in RegulonDB	73	105
Single or fist gene	61	59
Average 5‘-UTR length [nt]	81	121
Distal in polycistronic transcript (Regulon DB)	12 (16%)	46 (44%)
Intergenic distance <15 nt (genome annotation)	13 (13%)	39 (28%)

However, it turned out that the fraction of genes that are downstream genes in polycistronic transcripts is much higher in the group of resistant than in the group of sensitive genes (Table 2). RegulonDB contains 105 of the resistant transcripts, 46% of which are noted to be localized as downstream genes on polycistronic transcripts, in contrast, this is only true for 16% of the 73 sensitive transcripts that are listed in RegulonDB. A small intergenic distance of less than 15 nt in the genome annotation was taken as an indication for the formation of a polycistronic transcript regardless of the occurrence of the gene in RegulonDG. 28% of the 137 resistant genes had such a small intergenic distance to the upstream gene, but only 13% of the 102 sensitive genes. Taken together, translational coupling might be a second and novel feature of transcripts associated with resistance to Kasugamycin, in addition to the lack of a 5’-UTR.

Taking a different perspective, all genes were retrieved from RegulonDB that are proposed to have leaderless transcripts, and their translational efficiencies according to the translatome analysis were tabulated (S4 Table). RegulonDB lists 77 transcripts with a 5’-UTR of four nt or less, and 97 transcripts with 5’-UTRs of seven nt or less (S4 Table). Only 16 of the 97 transcripts had been detected in the translatome analysis, indicating that the vast majority of 81 transcripts were either not present in cells in mid-exponential growth phase in complex medium, or their concentrations were so low that it was below the detection limit. In accordance with this view, for only 17 of the 97 genes transcripts could be observed in a transcriptome analysis (Lange, Schweitzer and Soppa, unpublished data).

For 14 of the 16 transcripts detected by the translatome analysis, RegulonDB lists more than one 5’-end and thus it is unclear whether the leadered or the leaderless version of the transcript was measured in the translatome analysis. Therefore, the determined low or average Kasugamycin resistances of these transcripts do not contradict the model that leaderless transcripts are resistant. Another transcript belongs to a downstream gene in an operon, and thus the tabulated leaderless version is probably a cleavage product and might be a degradation intermediate. This leaves a single gene with a single, leaderless transcript. It is the *torS* transcript that was found to have the highest Kasugamycin resistance of all 2801 transcripts analyzed (S1 Table). After a 10 minute Kasugamycin treatment, it has a 100-fold higher translational efficiency than the most sensitive gene.

Because only a minority of the leaderless transcripts tabulated in RegulonDB were expressed under optimal conditions in complex medium, a literature search was performed to identify specialized functions of cellular leaderless transcripts in *E*. *coli*. Unexpectedly, 28 of the 97 genes listed to have transcripts with a very short “5’-UTR” in RegulonDB turned out to be genes for regulatory RNAs (sRNAs), tRNAs, RNaseP-RNA, and RNAs of toxin-antitoxin systems (S4 Table). For many of the remaining 69 genes differential expression and/or biological function have not been published, therefore, specific conditions that should allow the experimental validation of a large number of leaderless Kasugamycin resistant cellular *E*. *coli* transcripts could not be identified.

## Discussion

### The effects of Kasugamycin on *E*. *coli*

Kasugamycin is long known as an inhibitor of translation. More specifically, it was found to bind to the 30S and 70S, but not to the 50S ribosome, to inhibit the initiation step of translation, and to prevent the initiator tRNA to bind to the P-site of the ribosome [7]. Of course, inhibition of translation leads to subsequent effects on additional biological functions, and the categories “direct effects”, “indirect effects”, “secondary effects”, and “bystander effects” have been proposed [31]. It has been shown that a 30 minute Kasugamycin treatment of *E*. *coli* does not only influence translation, but has a considerably effect on the transcriptome and changed the transcript levels of several hundred genes [27]. After a 90 minutes treatment with Kasugamycin the occurrence of aberrant 61S ribosomes was observed, which lacked several proteins and could translate leaderless transcripts, but lost the ability to translate leadered transcripts [28]. As subsequent effects might overwhelm and hide the direct effects, a short-term 10 minutes treatment was chosen in this study, with the aim maximize direct and minimize indirect effects. However, even after only 10 minutes not only translation was affected, but also changes in the transcriptome were observed (Lange, Schweitzer and Soppa, unpublished results). While the magnitude of the effects were smaller, the trends observed for the 10 min (our results) and the 30 min [27] treatment were similar, i.e. the levels of transcripts for ribosomal and other translational proteins increased, while transcript levels for enzymes of the central metabolism decreased. While the Kasugamycin effects on the transcriptome were similar after 10 minutes and 30 minutes, there was no correlation between the transcriptome changes after a 10 minutes treatment compared to a 120 minutes treatment (Lange, Schweitzer and Soppa, unpublished results).

It is unclear whether the transcriptome changes after a 10 minutes treatment already represent subsequent down-stream effects of inhibiting translation initiation, or whether it is a direct effect of binding of Kasugamycin to one or more additional targets in the cell. It has been proposed that most antibiotics do not only bind to the target for which they are well-known, but that they have additional targets in the cell [31]. Because the inhibition of additional direct targets might have subsequent down-stream effects on translation initiation, the possibility that several targets might exist underscores the importance of short-term analyses, which concentrate on the elucidation of direct effects.

### The search for features determining Kasugamycin resistance or sensitivity

The structures of complexes comprised of Kasugamycin bound to either the 30S ribosomal subunit of *T*. *thermophilus* or to the 70S ribobome of *E*. *coli* have been determined [8,9]. Both studies were in agreement that the Kasugamycin binding site does not overlap with the binding site of the tRNA_i_, but that Kasugamycin binds to an upstream site, overlapping with the E site of the ribosome. These results rationalized earlier knowledge that Kasugamycin does not inhibit translation initiation on leaderless transcripts, or, to be exact, translation initiation on the leaderless phage transcript cI (often analyzed in fusion to a reporter transcript).

The results of the two structural studies also led to the prediction that the positions -3 to +1 of mRNAs would be of crucial importance for the sensitivity or resistance of a leadered transcript toward Kasagamycin [9]. In addition, it was reported that the presence of a strong SD motif resulted in Kasugamycin-resistance of the respective transcript [8]. While these results are true for the few specific transcripts investigated, the translatome analysis has revealed that they cannot be extrapolated to general rules. The RNA Structure Logos of the groups of Kasugamycin-sensitive and—resistant genes did not reveal any specific sequence dependence of the region -3 to -1. Furthermore, the presence of a strong SD motif does not seem to be a general determinant of Kasugamycin resistance, because the SD motif was retrieved with an extremely low information content from the group of resistant genes, and the SD motif was also retrieved from the group of sensitive genes. Therefore, while the X-ray structures correctly describe the Kasugamycin binding site on the 30S subunit of the ribosome, they cannot explain general features that result in sensitivity or resistance of transcripts towards the antibiotic in the cytoplasm of *E*. *coli*.

Analyses of the groups of resistant and sensitive genes with gene features tabulated in RegulonDB did not unravel any correlation between the degree of resistance and most features like 5’-UTR length, GC content, biological function, etc. Unexpectedly, the fraction of genes that are downstream genes on polycistronic transcripts (RegulonDB) was much higher in the group of resistant genes (46%) than in the group of sensitive genes (16%). Similarly, 28% of the resistant genes had a small intergenic distance to the upstream gene in the genome annotation, but only 13% of the sensitive genes. Therefore, translational coupling might be a novel mechanism leading to Kasugamycin resistance. Translational coupling means that translation of a downstream gene depends (exclusively or to a certain degree) on the translation of an upstream gene, and this coupling can be caused by different molecular mechanisms. One mechanism rests on the formation of a stem-loop structure between the upstream region of the downstream gene and parts of the open reading frame of the upstream gene, thereby occluding the SD motif of the downsteam gene. Translation of the upstream gene destroys this stem-loop and liberates the SD motif, thus enabling novel translation initiation by new ribosomes at the downstream gene. This mechanism has been described to operate at the *atpHA* gene pair of the operon encoding the ATP synthase [32]. Translational coupling has also been described for three other gene pairs of the *atp* operon, and the tightness of coupling followed the order *atpHA* > *atpFH* > *atpEF* > *atpAG* [33]. The KEV value for three of the downstream genes have been quantified, they were 1.44 for *atpA*, 1.76 for *atpF*, and 2.47 for *atpG*. At least *atpG* belongs to the resistant genes, however, the KEV values of the other two genes are not very high and there is no correlation to the tightness of coupling. A second mechanism of translational coupling on is named “termination-reinitiation” for eukaryotic viruses [34]. It requires an overlap of or a very short distance between the two coupled genes. The small subunit of the ribosome that had translated the upstream gene remains bound to the transcript and initiates translation at the nearby downstream gene. This mechanism has been proposed to operate also in *E*. *coli*, e.g. *trpB* and *trpA* have overlapping stop/start codons and this proximity is required for efficient coupling [35]. The *E*. *coli* genome contains several hundred overlapping gene pairs, and thus this mechanism of translational coupling vie termination-reinitiation might be quite common. Experimentally it has been shown to occur at five additional *E*. *coli* gene pairs with overlapping stop/start codons (Huber and Soppa, unpublished results). In addition, it has been proposed that the ribosome (or the small subunit) can scan the transcript in both directions after termination and restarts at the nearest native or artificially introduced start codon [36]. It is an attractive hypothesis that reinitiating ribosomes, which had not left the transcript after termination, might have a higher Kasugamycin resistance compared to cytoplasmic small subunits that are involved in novel initiation. The small distance between several resistant genes and their upstream genes (Table 2) might hint in that direction. Unfortunately, apart from *atpG* the translation of none of the resistant operon-bound genes has been studied, and thus future experiments are required to clarify whether one or both of the discussed mechanisms of translational coupling can lead to Kasugamycin resistance. However, translational coupling cannot explain the resistance of the majority of transcripts. Thus it seems that not only the absence of a 5’-UTR and possibly translational coupling, but also further molecular mechanisms can lead to Kasugamycin resistance.

### The scarcity of leaderless transcripts in *E*. *coli*

It is well documented that leaderless transcripts are resistant to the inhibition by Kasugamycin [8,9,11,37]. Therefore, it was anticipated that the group of resistant genes should be dominated or at least enriched in leaderless transcripts. Indeed, the transcript with the highest resistance, the *torS* transcript, is leaderless according to RegulonDB. However, this is the only leaderless transcript in the group of 135 resistant genes. A recent analysis of the *E coli* transcriptome led to the identification of five leaderless transcripts [38]. Three of these transcripts were derived from prophages, and only two belonged to cellular transcripts, i.e. *pgpA* encoding phosphatidylglycerophosphatase A and *rhoB* encoding a helicase.

Taken together, our translatome analysis and a transcriptome analysis [38] led to the experimental verification of only three cellular leaderless transcripts. However, both studies analyzed *E*. *coli* cultures grown under optimal conditions, and leaderless transcripts might prevail under different conditions, e.g. during starvation or under stress adaptation. To identify further leaderless *E*. *coli* transcripts, the database RegulonDB was analyzed. RegulonDB lists 77 transcripts with a 5’-UTR of four nt or less, and 97 transcripts with 5’-UTRs of seven nt or less (S4 Table). Only 16 of the 97 transcripts had been detected in the translatome analysis, indicating that the vast majority of 81 transcripts were not present in cells in mid-exponential growth phase in complex medium. In accordance with this view, for only 17 of the 97 genes transcripts were observed in the transcriptome analysis (Lange, Schweitzer, and Soppa, unpublished results). It remains to be discovered under which conditions the majority of genes with leaderless transcripts are induced.

Unexpectedly, 28 of the genes listed to have transcripts with a very short “5’-UTR” in RegulonDB turned out to be genes for regulatory RNAs, tRNAs, RNaseP-RNA, and RNAs of toxin-antitoxin systems. This reduces the maximal number of leaderless RNAs of protein-coding genes of *E*. *coli* to 69. This very small fraction of leaderless transcripts in *E*. *coli* (maximally 1.7%) is in contrast to other species of bacteria, archaea, and eukaryotes, e.g. in haloarchaea 72% of the transcripts are leaderless [13], in the bacteria *Mycobacterium tuberculosis*, *C*. *glutamicum*, and *D*. *deserti* and 26%, 33%, and 47%, respectively, of all primary transcripts are leaderless [21–23], and in the eukaryotes *Giardia lamblia* all transcripts are leaderless [20]. Therefore, *E*. *coli* does not seem to be the best species to characterize the mechanism of translation initiation on leaderless transcripts, as results obtained with a species that hardly makes use of leaderless transcripts might not be representative. *E*. *coli* had been chosen for this study because the molecular mechanism of Kaugamycin has been studied nearly exclusively with this species. In the future, archaeal or bacterial model species with a high fraction of leaderless transcriptsshould also be used to characterize the effects of Kasugamycin, and specifically, whether all leaderless transcripts are Kasugamycin-resistant.

### Conclusions

Kasugamycin does not only influence translation, but—directly or indirectly—also leads to transcriptome changes. The results of the translatome analysis are not in accordance with previous predictions that the positions -3 to -1 upstream of the start codon and an extended Shine Dalgarno motif are crucial for the Kasugamycin resistance of transcripts. Instead, the group of Kasugamycin resistant transcripts is enriched in downstream genes of polycistronic operons with a short distance to the upstream gene. Therefore, translational coupling seems to be a novel mechanism of Kasugamycin resistance, which has not been discussed before. RNA Structure Logos did not reveal any significant sequence motif diversity between sensitive and resistant genes, therefore, various individual transcript features seem to be compatible with Kasugamycin resistance. The transcript with the highest resistance was leaderless, in accordance with previous predictions, but it was the only leaderless transcript that could be detected. This translatome study, a transcriptome study, and the database RegulonDB indicate that the number of leaderless *E*. *coli* transcripts is extremely small, in contrast to other species of bacteria and archaea, which contain high fractions of leaderless transcripts.

## Materials and Methods

### *E*. *coli* strain and media

The *E*. *coli* strain MG1655 was kindly provided by Isabella Moll (University of Vienna, Austria). It was cultivated in LB complex medium under standard conditions.

### Translatome analysis

The tranlatome analyses with *E*. *coli* were performed similar to the translatome analyses with two species of haloarchaea described previously [39]. Each translatome analysis was started with a freshly grown *E*. *coli* colony that was used to inoculate a 5 ml overnight culture. An aliquot was diluted 100-fold into fresh medium and cultivated for 2 h to generate an exponentially growing culture. This was used to inoculate 150 ml medium with an OD_600_ of 0.02. The culture was incubated until it reached an OD_600_ of 0.2. 30 ml were removed as an untreated control. Kasugamycin was added to the culture (750 μg/ml), and a further 30 ml aliquot was removed after ten minutes incubation. Immediately after removal the aliquots were added to 25 g ice pre-cooled to -20°C. Cells were harvested by centrifugation and resuspended in 350 μl lysis buffer (10 mM Tris/HCl pH 7.4, 10 mM magnesium acetate, 60 mM NH_4_Cl, 6 mM beta-mercaptoethanol). Cell were lysed by sonication, and cell debris was removed by centrifugation.

Aliquots of cell extracts with an OD_260_ of 10 were layered onto sucrose density gradients ranging from 10% (w/w) to 40% (w/w) sucrose. Centrifugation was performed in a SW40 swing-out rotor at 17 000 rpm for 19 h. The gradients were fractionated using Teledyne 160 gradient harvester in fractions of about 0.7 ml volume. The OD_260_ curves were used to identify fractions with free RNA and with polysomal RNA, respectively, which were pooled separately. RNA isolation, cDNA synthesis, and DNA microarray analysis were performed as described previously [40, 41]. The analyses were performed separately for Kasugamycin-treated and for mock treated cultures. In each case, five biological replicates were performed, including a dye swap for two replicates. The translational efficiencies were calculated as the quotients of the values for the polysomal and the free RNAs. Average values, standard variations and variation coefficients were calculated. As the variance of translatome analyses can be larger than that of transcriptome analysis, the Excel table was sorted according to the variation coefficients, both for the Kasugamycin-treated and mock-treated cultures. The results with variation coefficients of 60% and higher were visually inspected, and obvious outliers were removed from the analysis. Lastly, the degree of the Kasugamycin effect on the translational efficiency was calculated as the quotient of the results of the Kasugamycin-treated and the mock-treated culture.

### Databases and bioinformatic analyses

The following databases were used to address the *E*. *coli* genome sequence and retrieve sequences: EcoGene2 (www.ecogene) [42] and Halolex (www.halolex.mpg.de) [43]. The database RegulonDG (regulondb.ccg.unam.mx) was used to retrieve selected features of *E*. *coli* genes, e.g. the lengths of 5’-UTRs and the genomic localization. The presence of possible sequence motifs in groups of genes was analyzed using RNA Structure Logo (www.cbs.dtu.dk/~gorodkin/appl/slogo.html) [44] for motifs with a fixed distance to the translation start codon and with MEME (meme.nbcr.net/meme) [45] for motifs with a variable distance to the start codon. For the generation of RNA Structure LOGOs the respective fractions of the four nucleotides were calculated for each set of genes and used to determine deviations from statistical expectations. They were quite similar for the five analyzed gene sets and had values of 0.28–0.30 for A, 0.22–0.24 for G, 0.21–0.23 for C, and 0.25–0.27 for T.

## Supporting Information

S1 Fig RNA Structure Logo of 100 “rather sensitive genes” with KEV from 0.51 to 0.61. For further explanations see the Legend of Fig 4.
(PPT)

S2 Fig RNA Structure Logo of all 97 “very average genes” with KEV from 0.99 to 1.01. For further explanations see the Legend of Fig 4.
(PPT)

S3 Fig RNA Structure Logo of 100 “rather resitant genes” with KEV from 1.80 to 1.99. For further explanations see the Legend of Fig 4.
(PPT)

S1 Table Summary of results of comparing the translatomve of *E*. *coli* in the presence and absence of Kasugamycin. Listed are the gene names, annotation, results of the five DNA microarray analyses in the absence and presence of Kasugamycin, respectively, average results, KEV values, and the p-values of a t-test.
(XLS)

S2 Table List of genes that are translationally down-regulated in the presence of Kasugamycin. (XLS)

S3 Table Resistant transcripts (KEV >2.0) and selected features taken from RegulonDB. (XLS)

S4 Table List of all genes that are listed in RegulonDB to be leaderless (5‘-UTRs of 7 nt or less) and selected features. (DOC)

## References

[1] Umezawa H, Hamada M, Suhara Y, Hashimoto T, Ikekawa T (1965) Kasugamycin, a new antibiotic. Antimicrob Agents Chemother (Bethesda) 5: 753–757. 5883494

[2] Wade L, Forster H, Adaskaveg J (2012) Kasugamycin and kasugamycin-fungicide mixtures for managing bacterial spot of tomato. Phytopathology 102: 129–129.

[3] Kansara S, Joshi D, Dhingani J (2012) Management of Leaf Spot/Blight of Heliconia Caused by [Drechslera State of *Trichometasphaeria holmii]* Using Fungicides. J Pure Appl Microbio 6: 1715–1719.

[4] Adaskaveg J, Forster H, Wade L (2010) Re-discovery of kasugamycin for managing fire blight and other bacterial diseases of plants in the United States. Phytopathology 100: S166.

[5] Vallad G, Pernezny K, Balogh B, Wen A, Figueiredo J, Jones J, et al (2010) Comparison of Kasugamycin to traditional bactericides for the management of bacterial spot on tomato. Hortscience 45: 1834–1840.

[6] Ishigami J, Fukuda Y, Hara S (1967) Clinical use of Kasugamycin for urinary tract infections due to *Pseudomonas aeruginosa*. J Antibiot B 20: 83–84. 5299159

[7] Okuyama A, Tanaka N, Komai T (1975) The binding of kasugamycin to the *Escherichia coli* ribosomes. J Antibiot (Tokyo) 28: 903–905. 1104550 10.7164/antibiotics.28.903

[8] Schluenzen F, Takemoto C, Wilson DN, Kaminishi T, Harms JM, Hanawa-Suetsugu K, et al (2006) The antibiotic kasugamycin mimics mRNA nucleotides to destabilize tRNA binding and inhibit canonical translation initiation. Nat Struct Mol Biol 13: 871–878. 10.1038/nsmb1145 16998488

[9] Schuwirth BS, Day JM, Hau CW, Janssen GR, Dahlberg AE, Cate JH, et al (2006) Structural analysis of kasugamycin inhibition of translation. Nat Struct Mol Biol 13: 879–886. 10.1038/nsmb1150 16998486 PMC2636691

[10] Moll I, Blasi U (2002) Differential inhibition of 30S and 70S translation initiation complexes on leaderless mRNA by kasugamycin. Biochem Biophys Res Commun 297: 1021–1026. 12359258 10.1016/s0006-291x(02)02333-1

[11] Moll I, Hirokawa G, Kiel MC, Kaji A, Blasi U (2004) Translation initiation with 70S ribosomes: an alternative pathway for leaderless mRNAs. Nucleic Acids Res 32: 3354–3363. 10.1093/nar/gkh663 15215335 PMC443539

[12] Krishnan KM, Van Etten WJ 3rd, Janssen GR (2010) Proximity of the start codon to a leaderless mRNA's 5' terminus is a strong positive determinant of ribosome binding and expression in *Escherichia coli*. J Bacteriol 192: 6482–6485. 10.1128/JB.00756-10 20971908 PMC3008523

[13] Babski J, Haas KA, Näther-Schindler D, Pfeiffer F, Förstner KU, Hammelmann M, et al (2016) Genome-wide Identification of transcriptional start sites in the haloarchaeon *Haloferax volcanii* based on differential RNA-Seq (dRNA-Seq). BMC Genomics 17: 629 10.1186/s12864-016-2920-y 27519343 PMC4983044

[14] Hering O, Brenneis M, Beer J, Suess B, Soppa J (2009) A novel mechanism for translation initiation operates in haloarchaea. Mol Microbiol 71: 1451–1463. 10.1111/j.1365-2958.2009.06615.x 19210623

[15] Andreev DE, Terenin IM, Dunaevsky YE, Dmitriev SE, Shatsky IN (2006) A leaderless mRNA can bind to mammalian 80S ribosomes and direct polypeptide synthesis in the absence of translation initiation factors. Mol Cell Biol 26: 3164–3169. 10.1128/MCB.26.8.3164-3169.2006 16581790 PMC1446950

[16] Grill S, Moll I, Giuliodori AM, Gualerzi CO, Blasi U (2002) Temperature-dependent translation of leaderless and canonical mRNAs in *Escherichia coli*. FEMS Microbiol Lett 211: 161–167. 12076807 10.1111/j.1574-6968.2002.tb11219.x

[17] Moll I, Grill S, Gualerzi CO, Blasi U (2002) Leaderless mRNAs in bacteria: surprises in ribosomal recruitment and translational control. Mol Microbiol 43: 239–246. 11849551 10.1046/j.1365-2958.2002.02739.x

[18] Brock JE, Pourshahian S, Giliberti J, Limbach PA, Janssen GR (2008) Ribosomes bind leaderless mRNA in *Escherichia coli* through recognition of their 5'-terminal AUG. RNA 14: 2159–2169. 10.1261/rna.1089208 18755843 PMC2553737

[19] Van Etten WJ, Janssen GR (1998) An AUG initiation codon, not codon-anticodon complementarity, is required for the translation of unleadered mRNA in *Escherichia coli*. Mol Microbiol 27: 987–1001. 9535088 10.1046/j.1365-2958.1998.00744.x

[20] Li L, Wang CC (2004) Capped mRNA with a single nucleotide leader is optimally translated in a primitive eukaryote, *Giardia lamblia*. J Biol Chem 279: 14656–14664. 10.1074/jbc.M309879200 14722094

[21] Cortes T, Schubert OT, Rose G, Arnvig KB, Comas I, Aebersold R, et al (2013) Genome-wide mapping of transcriptional start sites defines an extensive leaderless transcriptome in *Mycobacterium tuberculosis*. Cell Rep 5: 1121–1131. 10.1016/j.celrep.2013.10.031 24268774 PMC3898074

[22] Pfeifer-Sancar K, Mentz A, Ruckert C, Kalinowski J (2013) Comprehensive analysis of the *Corynebacterium glutamicum* transcriptome using an improved RNAseq technique. BMC Genomics 14: 888 10.1186/1471-2164-14-888 24341750 PMC3890552

[23] de Groot A, Roche D, Fernandez B, Ludanyi M, Cruveiller S, Pignol D, et al (2014) RNA sequencing and proteogenomics reveal the importance of leaderless mRNAs in the radiation-tolerant bacterium *Deinococcus deserti*. Genome Biol Evol 6: 932–948. 10.1093/gbe/evu069 24723731 PMC4007540

[24] Chang B, Halgamuge S, Tang SL (2006) Analysis of SD sequences in completed microbial genomes: non-SD-led genes are as common as SD-led genes. Gene 373: 90–99. 10.1016/j.gene.2006.01.033 16574344

[25] Shultzaberger RK, Bucheimer RE, Rudd KE, Schneider TD (2001) Anatomy of *Escherichia coli* ribosome binding sites. J Mol Biol 313: 215–228. 10.1006/jmbi.2001.5040 11601857

[26] Hayashi K, Morooka N, Yamamoto Y, Fujita K, Isono K, Choi S, et al (2006) Highly accurate genome sequences of *Escherichia coli* K-12 strains MG1655 and W3110. Mol Syst Biol:. 10.1038/msb4100049 PMC1681481 16738553

[27] Sabina J, Dover N, Templeton LJ, Smulski DR, Soll D, LaRossa RA (2003) Interfering with different steps of protein synthesis explored by transcriptional profiling of *Escherichia coli* K-12. J Bacteriol 185: 6158–6170. 10.1128/JB.185.20.6158-6170.2003 14526028 PMC225041

[28] Kaberdina AC, Szaflarski W, Nierhaus KH, Moll I (2009) An unexpected type of ribosomes induced by kasugamycin: a look into ancestral times of protein synthesis? Mol Cell 33: 227–236. 10.1016/j.molcel.2008.12.014 19187763 PMC2967816

[29] Mendoza-Vargas A, Olvera L, Olvera M, Grande R, Vega-Alvarado L, Taboada B, et al (2009) Genome-wide identification of transcription start sites, promoters and transcription factor binding sites in *E*. *coli*. PLoS One 4: e7526 10.1371/journal.pone.0007526 19838305 PMC2760140

[30] Salgado H, Peralta-Gil M, Gama-Castro S, Santos-Zavaleta A, Muniz-Rascado L, Garcia-Sotelo JS, et al (2012) RegulonDB v8.0: omics data sets, evolutionary conservation, regulatory phrases, cross-validated gold standards and more. Nucleic Acids Res 41: D203–213. 10.1093/nar/gks1201 23203884 PMC3531196

[31] Brazas MD, Hancock RE (2005) Using microarray gene signatures to elucidate mechanisms of antibiotic action and resistance. Drug Discov Today 10: 1245–1252. 10.1016/S1359-6446(05)03566-X 16213417

[32] Rex G, Surin B, Besse G, Schneppe B, McCarthy JE (1994) The mechanism of translational coupling in *Escherichia coli*. Higher order structure in the *atpHA* mRNA acts as a conformational switch regulating the access of *de novo* initiating ribosomes. J Biol Chem 269: 18118–18127. 7517937

[33] Hellmuth K, Rex G, Surin B, Zinck R, McCarthy JE (1991) Translational coupling varying in efficiency between different pairs of genes in the central region of the *atp* operon of *Escherichia coli*. Mol Microbiol 5: 813–824. 1830358 10.1111/j.1365-2958.1991.tb00754.x

[34] Powell ML (2010) Translational termination-reinitiation in RNA viruses. Biochem Soc Trans 38: 1558–1564. 10.1042/BST0381558 21118126

[35] Das A, Yanofsky C (1989) Restoration of a translational stop-start overlap reinstates translational coupling in a mutant trpB’-trpA gene pair of the *Escherichia coli* tryptophan operon. Nucleic Acids Res 17: 9333–9340. 2685759 10.1093/nar/17.22.9333 PMC335135

[36] Adhin MR, van Duin J (1990) Scanning model for translation reinitiation in eubacteria. J Mol Biol 231: 811–818. 10.1016/S0022-2836(05)80265-7 2193163

[37] Chin K, Shean CS, Gottesman ME (1993) Resistance of lambda cI translation to antibiotics that inhibit translation initiation. J Bacteriol 175: 7471–7473. 8226693 10.1128/jb.175.22.7471-7473.1993 PMC206893

[38] Romero DA, Hasan AH, Lin YF, Kime L, Ruiz-Larrabeiti O, Urem M, et al (2014) A comparison of key aspects of gene regulation in *Streptomyces coelicolor* and *Escherichia coli* using nucleotide-resolution transcription maps produced in parallel by global and differential RNA sequencing. Mol Microbiol,. 10.1111/mmi.12810 PMC4681348 25266672

[39] Lange C, Zaigler A, Hammelmann M, Twellmeyer J, Raddatz G, Schuster S, et al (2007) Genome-wide analysis of growth phase-dependent translational and transcriptional regulation in halophilic archaea. BMC Genomics 8: 415 10.1186/1471-2164-8-415 17997854 PMC3225822

[40] Veit A, Polen T, Wendisch VF (2007) Global gene expression analysis of glucose overflow metabolism in *Escherichia coli* and reduction of aerobic acetate formation. Appl Microbiol Biotechnol 74: 406–421. 10.1007/s00253-006-0680-3 17273855

[41] Wendisch VF, Zimmer DP, Khodursky A, Peter B, Cozzarelli N, Kustu S (2001) Isolation of *Escherichia coli* mRNA and comparison of expression using mRNA and total RNA on DNA microarrays. Anal Biochem 290: 205–213. 10.1006/abio.2000.4982 11237321

[42] Zhou J, Rudd KE (2012) EcoGene 3.0. Nucleic Acids Res 41: D613–624. 10.1093/nar/gks1235 23197660 PMC3531124

[43] Pfeiffer F, Broicher A, Gillich T, Klee K, Mejia J, Rampp M, et al (2008) Genome information management and integrated data analysis with HaloLex. Arch Microbiol 190: 281–299. 10.1007/s00203-008-0389-z 18592220 PMC2516542

[44] Gorodkin J, Heyer LJ, Brunak S, Stormo GD (1997) Displaying the information contents of structural RNA alignments: the structure logos. Comput Appl Biosci 13: 583–586. 9475985 10.1093/bioinformatics/13.6.583

[45] Bailey TL, Boden M, Buske FA, Frith M, Grant CE, Clementi L, et al (2009) MEME SUITE: tools for motif discovery and searching. Nucleic Acids Res 37: W202–W208. 10.1093/nar/gkp335 19458158 PMC2703892

