# Supplementary figures and images for: Effects of Kasugamycin on the Translatome of Escherichia coli
Source: PLoS One. 2017 Jan 12;12(1):e0168143. doi: 10.1371/journal.pone.0168143 (PMC5230787; doi:10.1371/journal.pone.0168143)

## Slide 1
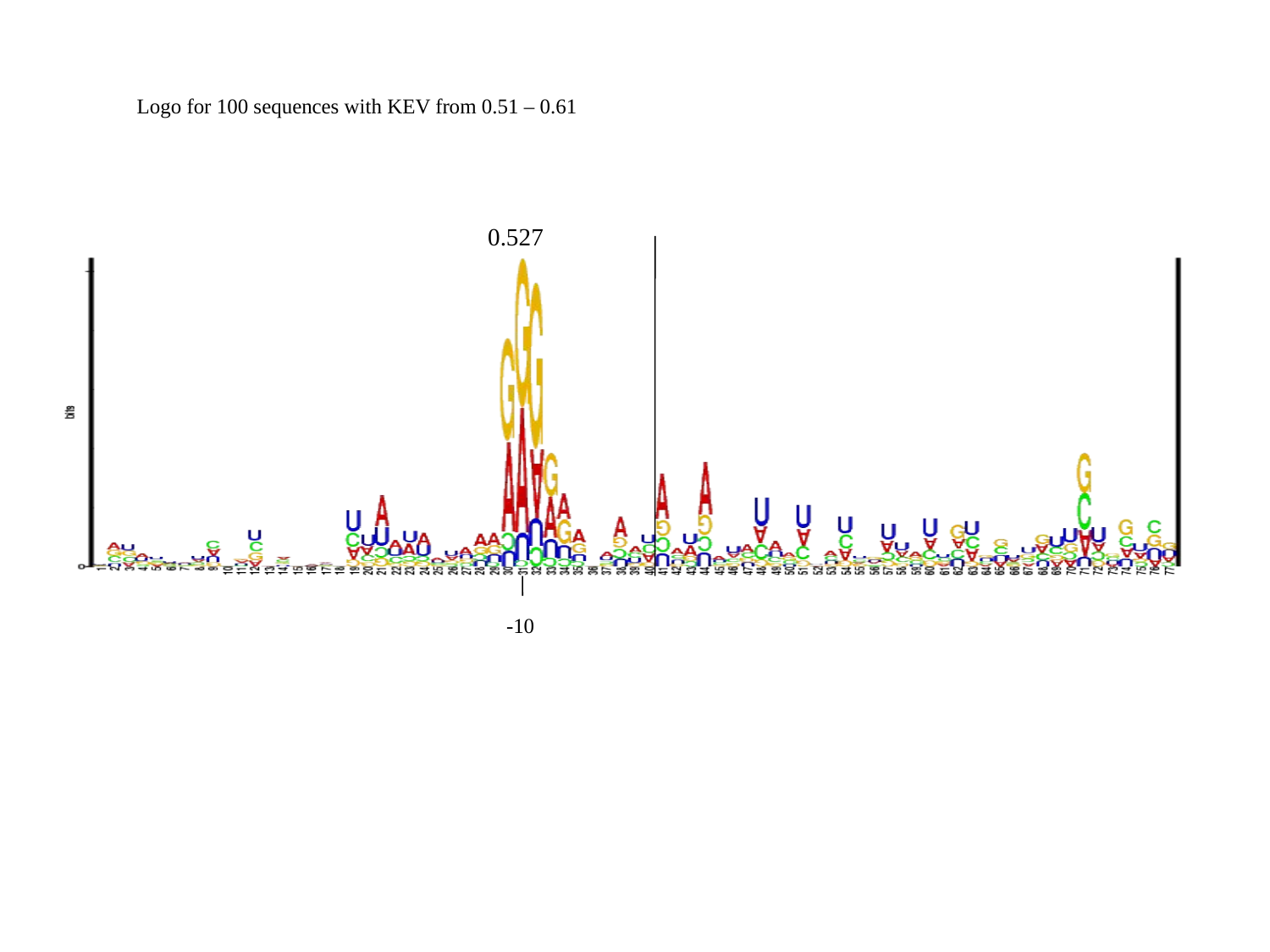

Logo for 100 sequences with KEV from 0.51 – 0.61
0.527
-10

Supplement: S1 Fig — For further explanations see the Legend of Fig 4. (PPT) [file pone.0168143.s001.ppt]

## Slide 1
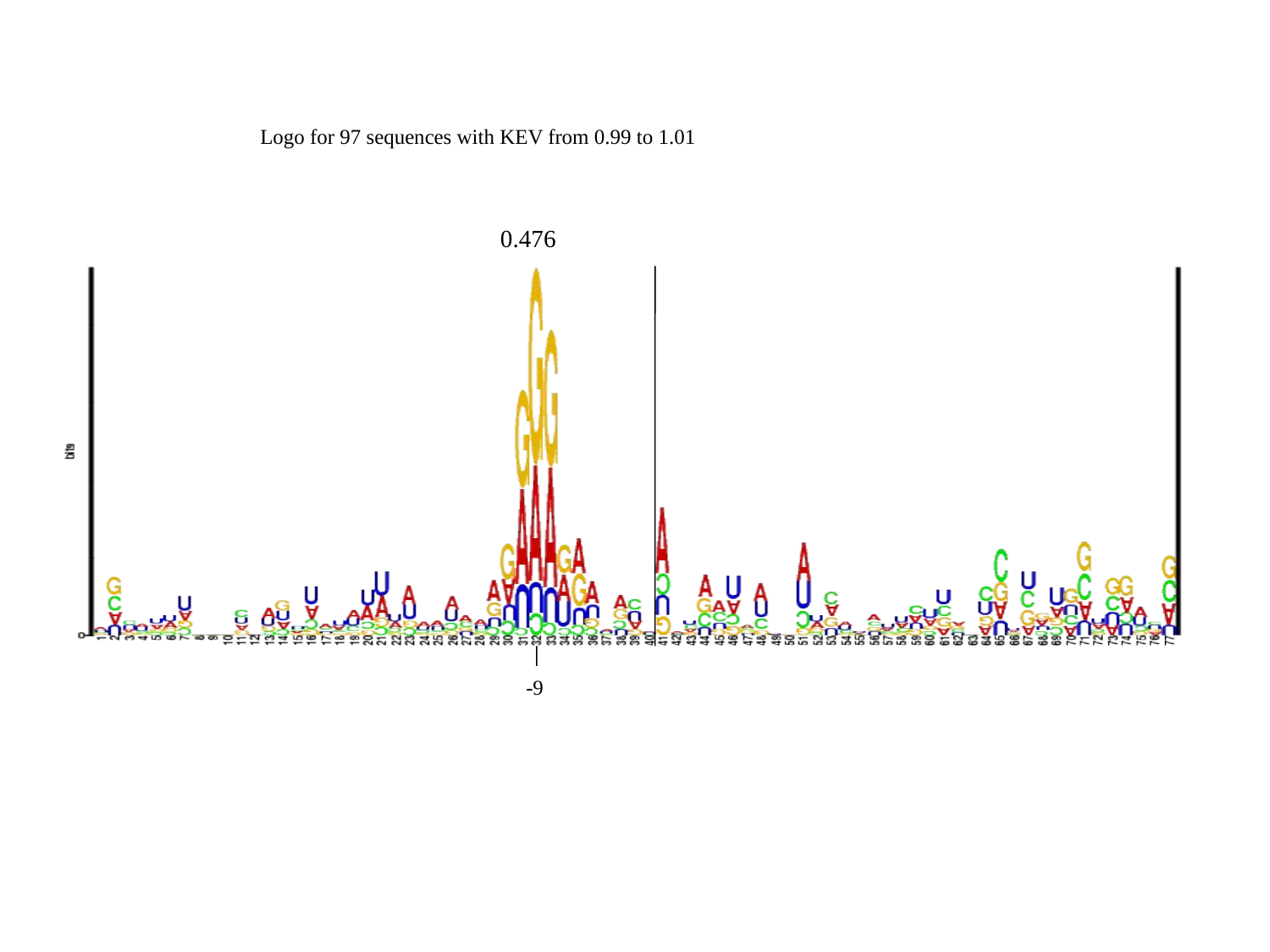

Logo for 97 sequences with KEV from 0.99 to 1.01
0.476
-9

Supplement: S2 Fig — For further explanations see the Legend of Fig 4. (PPT) [file pone.0168143.s002.ppt]

## Slide 1
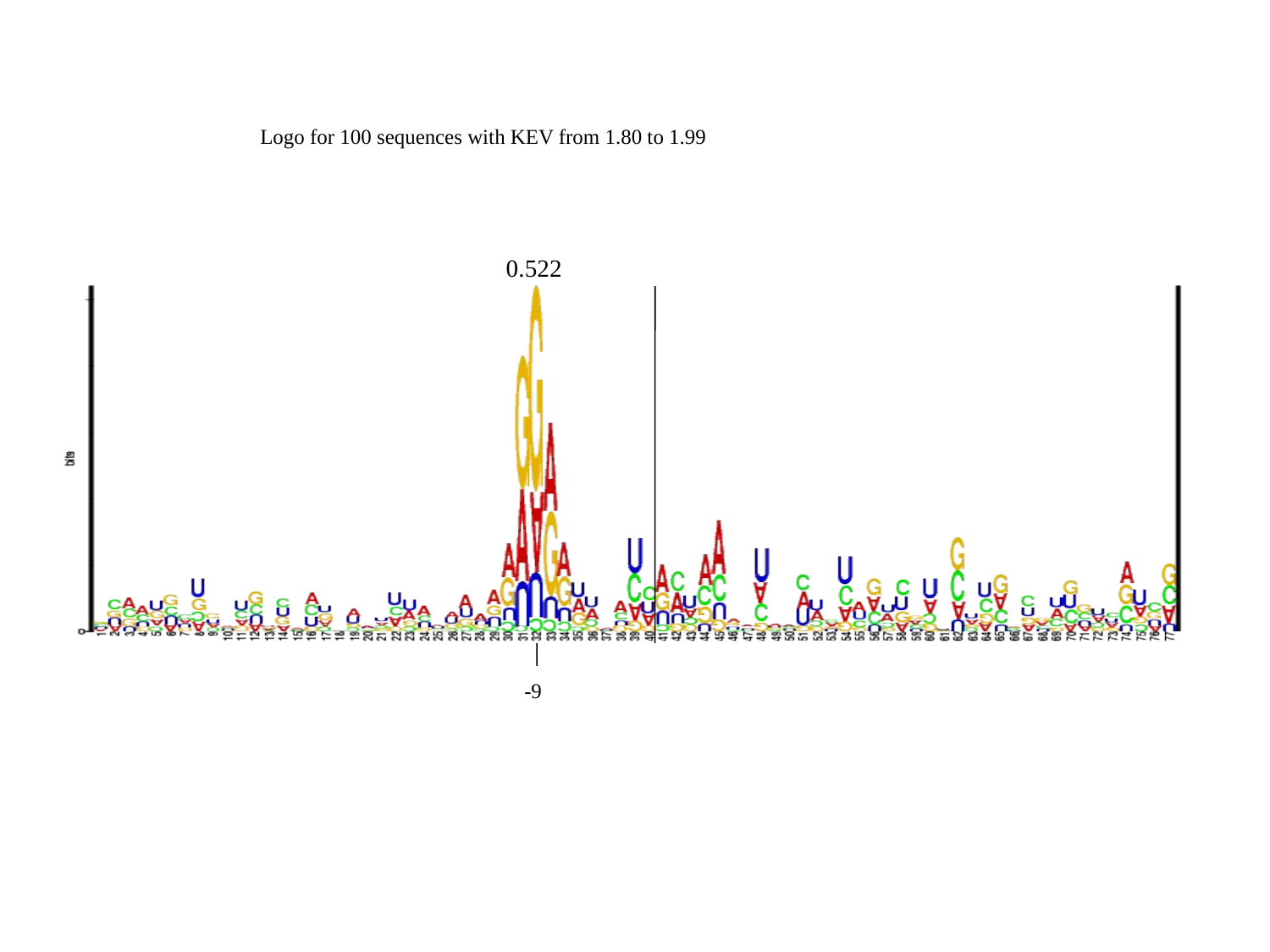

Logo for 100 sequences with KEV from 1.80 to 1.99
0.522
-9

Supplement: S3 Fig — For further explanations see the Legend of Fig 4. (PPT) [file pone.0168143.s003.ppt]
