# Supplementary material for: Effects of Kasugamycin on the Translatome of Escherichia coli
Source: PLoS One. 2017 Jan 12;12(1):e0168143. doi: 10.1371/journal.pone.0168143 (PMC5230787; doi:10.1371/journal.pone.0168143)
Supplement: S4 Table — (DOC) [file pone.0168143.s007.doc]

Supplementary Table S4. List of all genes that are listed in RegulonDB to be leaderless (5‘-UTRs of 7 nt or less) and selected features. The transcriptome results are unpublished data (Lange, Schweitzer, and Soppa)

| b-No. | Gene | 5'-UTR | Promoter No. | No. starts | Kasugamycin | Transkriptome | Non-coding | Gene |
| --- | --- | --- | --- | --- | --- | --- | --- | --- |
|  |  |  |  |  | resistance |  | RNA |  |
|  |  |  |  |  | [KEV] |  |  |  |
|  |  |  |  |  |  |  |  |  |
|  | sroA | G |  | 1 | no | - |  | sroA |
| 4577 | sgrS | G |  | 1 | no | - |  | sgrS |
| 0167 | glnD | GCACAA |  | 1 | no | - |  | glnD |
| 0169 | rpsB | C | aber: tff-rpsB-tsf | 1 | no | - |  | rpsB |
| 0174 | ispU | GCG | "P2" | 2 | 1,28 | + |  | ispU |
| 0240 | crl | TAGCAA | P2 | 2 | 1,98 | - |  | crl |
| 0375 | yaiV | A |  | 1 | no | - |  | yaiV |
| 0468 | ybaN | AATA | P9 | 4 | no | - |  | ybaN |
| 4585 | micM | A |  | 1 | no | - |  | micM |
| 0590 | fepDGC | CGTA | P2 | 2 | no | - |  | fepDGC |
| 0632 | dacA | GACCA | P2 | 2 | no | + |  | dacA |
| 0836 | bssR | GTA | P5 | 1 | no | - |  | bssR |
| 4417 | rybB | G |  | 1 | no | - | sRNA | rybB |
| 0881 | clpS | AA | P7 | 2 | 1,34 | + |  | clpS |
| 0968 | yccX | GCAA | P7 | 1 | no | - |  | yccX |
| 0993 | torS | GACCG | P5 | 1 | 5,86 | - |  | torS |
| 1018 | efeOB | A | P1 | 2 | no | - |  | efeOB |
| 1044 | ymdA | AAA | P9 | 3 | no | + |  | ymdA |
| 4418 | sraB | T |  | 1 | no | - |  | sraB |
| 1088 | yceD-rpmF | CCTA | P2 | 2 | 0,4 | + |  | yceD-rpmF |
| 4420 | rdlA | G | P5 | 1 | no | - |  | rdlA |
| 4422 | rdlB | GTG | P4 | 2 | no | - |  | rdlB |
| 4424 | rdlC | GTG | P3 | 2 | no | - |  | rdlC |
| 1229 | tpr | A | tyrTV-tpr | operon | 0,99 | + |  | tpr |
| 1276 | acnA | AGCTA | P3 | 3 | no | + |  | acnA |
| 4426 | mcaS | A |  | 1 | no | - | sRNA | mcaS |
| 4699 | fnrS | G |  | 1 | no | - |  | fnrS |
| 1356 | racR | AA | P5 | 2 | 0,51 | + |  | racR |
| 1356 | racR | GCAA | P3 | 2 | 0,51 |  |  | racR |
| 4427 | micC | G |  | 1 | no | - | sRNA | micC |
| 1414 | ydcF | AACCA | P4 | 2 | no | + |  | ydcF |
| 4597 | rydC | C |  | 1 | no | - |  | rydC |
| 4429 | sokB | ATTG | P3 | 1 | no | - |  | sokB |
| 1503 | ydeR | ATA | ydeTSR | 1 | no | - |  | ydeR |
| 4698 | mgrR | G |  | 1 | no | - |  | mgrR |
| 1598 | ydgD | GATA | P7 | 2 | 1,25 | - |  | ydgD |
| 1649 | nemRA | GCA |  | 1 | no | - |  | nemRA |
| 4431 | rprA | A |  | 1 | no | - | sRNA | rprA |
| 1769 | ydjE | CCTAA | P5 | 2 | 1,06 | - |  | ydjE |
| 4432 | ryeA | A |  | 1 | no | - |  | ryeA |
| 1954 | dsrA | A |  | 1 | no | - | sRNA | dsrA |
| 1932 | yedI | GGGAA | P3 | 2 | no | + |  | yedI |
| 4603 | rseX | T |  | 1 | no | - |  | rseX |
| 1975 | serU | TGAACG |  | 1 | no | - |  | serU |
| 1986 | asnU | AACGATT |  | 1 | no | - |  | asnU |
| 2037 | rfbX | GCA | P6 | 4 | 0,93 | + |  | rfbX |
| 4436 | sibA | G |  | 1 | no | - |  | sibA |
| 4437 | sibB | G |  | 1 | no | - |  | sibB |
| 4438 | cyaR | G |  | 1 | no | - | sRNA | cyaR |
| 2189 | proL | GAGTC |  | 1 | no | - |  | proL |
| 4439 | micF | G |  | 1 | no | - | sRNA | micF |
| 2348 | argW | TG |  | 1 | no | - |  | argW |
| 4441 | glmY | A |  | 1 | no | - | sRNA | glmY |
| 4608 | ohsC | TTG |  | 1 | no | - | antitoxin | ohsC |
| 4609 | ryfD | A |  | 1 | no | - |  | ryfD |
| 2602 | yfiL | AAGAAA | P10 | 2 | 0,87 | - |  | yfiL |
| 4442 | micA | G |  | 1 | no | - | sRNA | micA |
| 4408 | csrB | G |  | 1 | no | - | sRNA | csrB |
| 4443 | gcvB | A |  | 1 | no | - | sRNA | gcvB |
| 2814 | metZ | ACGGAC |  | 1 | no | - | tRNA | metZ |
| 4444 | omrA | C |  | 1 | no | - |  | omrA |
| 4445 | omrB | C |  | 1 | no | - |  | omrB |
| 4446 | sibC | A |  | 1 | no | - |  | sibC |
| 2959 | yggI | GCCGCTA | P5 | 4 | 1,12 | - |  | yggI |
| 2959 | yggI | GCTA | P4 | 4 | 1,12 | - |  | yggI |
| 2967 | pheV | TG |  | 1 | no | - | tRNA | pheV |
|  | sroG | G |  | 1 | no | - |  | sroG |
| 4447 | sibD | A |  | 1 | no | - |  | sibD |
|  | sibG | A |  | 1 | no | - |  | sibG |
| 3069 | ileX | TG |  | 1 | no | - | tRNA | ileX |
| 3123 | rnpB | G | garPLRK-rnpB | 1 | no | - | RNaseP | rnpB |
| 4450 | arcZ | G |  | 1 | no | - | sRNA | arcZ |
| 4451 | ryhB | G |  | 1 | no | - | sRNA | ryhB |
| 3488 | yhiJ | AAAATA | P6 | 2 | no | - |  | yhiJ |
| 4712 | agrA | T |  | 1 | no | - |  | agrA |
| 4713 | agrB | T |  | 1 | no | - |  | agrB |
| 4704 | arrS | G |  | 1 | no | - | sRNA | arrS |
| 4452 | gadY | A |  | 1 | no | - | sRNA | gadY |
| 4454 | rdlD | G |  | 1 | no | - |  | rdlD |
| 3545 | proK | TTC |  | 1 | no | - |  | proK |
| 3585 | yiaU | GTTGCGA | P8 | 2 | 1,13 | + |  | yiaU |
| 4616 | istR-1 | G |  | 1 | no | - | sRNA | istR-1 |
| 4616 | istR-2 | G |  | 1 | no | - | sRNA | istR-2 |
| 3718 | yieK | ATTAAGA | bglH-yieLK | 2 | 0,46 | + |  | yieK |
| 4456 | glmZ | G |  | 1 | no | - | sRNA | glmZ |
| 3822 | recQ | GTGAATG |  | 2 | 1,91 | + |  | recQ |
| 4707 | esrE | C |  | 1 | no | - | sRNA | esrE |
| 3864 | spf | G |  | 1 | 1,02 | - | sRNA | spf |
| 4457 | csrC | A |  | 1 | no | - | sRNA | csrC |
| 3885 | yihX | CAAAAA |  | 1 | no | + |  | yihX |
| 4458 | oxyS | G |  | 1 | no | - | sRNA | oxyS |
| 4691 | sroH | G |  | 1 | no | - |  | sroH |
| 4031 | xylE | GTCTGAA | P8 | 2 | 0,75 | + |  | xylE |
| 4459 | ryjA | A |  | 1 | no | - |  | ryjA |
| 4130 | yjdL | ACA | P2 | 4 | no | - |  | yjdL |
| 4134 | pheU | TG |  | 1 | no | - | tRNA | pheU |
| 4218 | ytfI | GTCATA | P9 | 4 | no | + |  | ytfI |
| 4624 | ryjB | T |  | 1 | no | - |  | ryjB |
| 4625 | symR | A |  | 1 | no | - | antitoxin | symR |
